# Identification of the hub gene BUB1B in hepatocellular carcinoma via bioinformatic analysis and in vitro experiments

**DOI:** 10.7717/peerj.10943

**Published:** 2021-02-23

**Authors:** Jie Fu, Xiao Zhang, Likun Yan, Yaoli Shao, Xinxu Liu, Yuan Chu, Ge Xu, Xundi Xu

**Affiliations:** 1 Department of General Surgery, The Second Xiangya Hospital, Central South University, Changsha, Hunan, China; 2 Hunan Provincial Key Laboratory of Hepatobiliary Disease Research, The Second Xiangya Hospital, Central South University, Changsha, Hunan, China

**Keywords:** Hepatocellular carcinoma, Bioinformatic analysis, Hub genes, BUB1B, In vitro

## Abstract

**Background:**

Hepatocellular carcinoma (HCC) is one of the most commonly diagnosed cancers and the fourth leading cause of cancer-related deaths in the world. Although the treatment of HCC has made great progress in recent years, the therapeutic effects on HCC are still unsatisfactory due to difficulty in early diagnosis, chemoresistance and high recurrence rate post-surgery.

**Methods:**

In this study, we identified differentially expressed genes (DEGs) based on four Gene Expression Omnibus (GEO) datasets (GSE45267, GSE98383, GSE101685 and GSE112790) between HCC and normal hepatic tissues. A protein–protein interaction (PPI) network was established to identify the central nodes associated with HCC. Gene Ontology (GO) and Kyoto Encyclopedia of Genes and Genomes (KEGG) analysis of the central nodes were conducted to find the hub genes. The expression levels of the hub genes were validated based on the ONCOMINE and Gene Expression Profiling Interactive Analysis (GEPIA) databases. Additionally, the genetic alterations of the hub genes were evaluated by cBioPortal. The role of the hub genes on the overall survival (OS) and relapse survival (RFS) of HCC patients was evaluated by Kaplan-Meier plotter. At last, the mechanistic role of the hub genes was illustrated by in vitro experiments.

**Results:**

We found the following seven hub genes: BUB1B, CCNB1, CCNB2, CDC20, CDK1, MAD2L1 and RRM2 using integrated bioinformatics analysis. All of the hub genes were significantly upregulated in HCC tissues. And the seven hub genes were associated with the OS and RFS of HCC patients. Finally, in vitro experiments indicated that BUB1B played roles in HCC cell proliferation, migration, invasion, apoptosis and cell cycle by partially affecting mitochondrial functions.

**Conclusions:**

In summary, we identified seven hub genes that were associated with the expression and prognosis of HCC. The mechanistic oncogenic role of BUB1B in HCC was first illustrated. BUB1B might play an important role in HCC and could be potential therapeutic targets for HCC.

## Introduction

Hepatocellular carcinoma (HCC), is one of the most commonly diagnosed cancers and the fourth leading cause of cancer-related deaths worldwide (Siegel, Miller & Jemal, 2017). Although the diagnosis and treatment of HCC have made great progress in recent years, the therapeutic effects of HCC are still unsatisfactory (Lee et al., 2020; Qin et al., 2020; Raoul & Edeline, 2020; Xia et al., 2019). Therefore, further study of the mechanisms underlying HCC development and metastasis is urgently needed, which will contribute to the development of treatment options and prolong survival for HCC patients.

Recently, microarrays coupled with bioinformatic analysis have been used to identify novel biomarkers related to carcinogenesis, tumor diagnosis, treatment and prognosis (Buisson et al., 2019; Emperle et al., 2019; Herrington et al., 2018). The major online databases include the Gene Expression Omnibus (GEO), European Genome-phenome Archive (EGA) and The Cancer Genome Atlas (TCGA) (Barrett et al., 2005; Cancer Genome Atlas Research et al., 2013; Lappalainen et al., 2015). In addition, online analysis websites such as ONCOMINE, cBioPortal, Kaplan Meier plotter and Gene Expression Profiling Interactive Analysis (GEPIA) have been used (Hou et al., 2017; Rhodes et al., 2007; Tang et al., 2019). It is useful for identifying the core genes related to HCC progression and prognosis by integrated bioinformatics analysis based on gene expression databases. At present, some core genes related to HCC have been found in some studies (Mi et al., 2020; Yang, Pan & You, 2019; Zhuang, Yang & Meng, 2018). However, the clinical sample size included in the analysis is not enough, and the mechanism of these genes in HCC has not been studied.

In this study, four mRNA microarray datasets (GSE45267, GSE98383, GSE101685 and GSE112790) were obtained from the GEO database (Diaz et al., 2018; Shimada et al., 2019; Wang et al., 2013). The online tool GEO2R was used to identify DEGs between HCC and normal hepatic tissues. Gene Ontology (GO) and Kyoto Encyclopedia of Genes and Genomes (KEGG) pathway enrichment analyses were conducted to further explore the functions of the DEGs (Huang da, Sherman & Lempicki, 2009). A protein-protein interaction (PPI) network was constructed to identify the hub genes associated with HCC via the Search Tool for the Retrieval of Interacting Genes (STRING) database (von Mering et al., 2003). Expression validation and survival analysis of the hub genes were conducted by Gene Expression Profiling Interactive Analysis (GEPIA), ONCOMINE, cBioPortal and Kaplan Meier plotter. Finally, the biological roles of the hub gene BUB1B in HCC were determined by in vitro experiments.

## Materials & Methods

### Microarray data

Gene expression profile data of HCC patients were obtained from the GEO database. The GSE45267, GSE98383, GSE101685 and GSE112790 datasets included 46 HCC tissues and 41 normal hepatic tissues, 11 HCC tissues and 27 normal hepatic tissues, 24 HCC tissues and eight normal hepatic tissues, and 183 HCC tissues and 15 normal hepatic tissues, respectively. All of the GEO datasets were based on the GPL570 platform.

### Identification of DEGs

DEGs between HCC tissues and normal hepatic tissues were identified via GEO2R online tools. Genes with —log2FC— > 2 and adjusted *P* value < 0.05 were considered DEGs. The intersecting genes of the four GEO datasets were examined via a Venn diagram. DEGs with log2FC < 2 were considered downregulated genes, while DEGs with log2FC > 2 were considered upregulated genes.

### Gene ontology annotation and KEGG pathway enrichment analysis

The functions of the DEGs were revealed by the online tool Database for Annotation, Visualization and Integrated Discovery (DAVID). Biological process (BP), cellular component (CC), molecular function (MF) and KEGG pathway analyses were conducted. Adj. *P* < 0.05 was considered statistically significant.

### PPI network construction and module analysis

The PPI network of significantly differentially expressed genes was constructed by the STRING database. All PPI pairs with a combined score of > 0.4 were extracted. The degrees of all nodes were calculated by the MCODE plugin in Cytoscape software.

### Validation of the expression levels of hub genes

To validate the mRNA expression levels of the identified hub genes between HCC and normal hepatic tissues, the ONCOMINE database and the online tool GEPIA were used.

### Genetic alterations and prognostic analysis of hub genes

The liver hepatocellular carcinoma (TCGA, Firehose Legacy), liver hepatocellular carcinoma (TCGA, PanCancer Atlas) (TCGA, Firehose Legacy), hepatocellular carcinomas (INSERM, Nat Genet 2015) and liver hepatocellular carcinoma (AMC, Hepatology 2014) datasets were used for the analysis of the genetic alterations of the hub genes with the online tool cBioPortal. The genetic alterations were categorized as missense mutations, truncating mutations, amplifications, deep deletions and no alterations. Kaplan Meier plots were used to compare the overall survival (OS) and disease-free survival (DFS) of HCC patients with or without alterations in the mRNA expression levels of the 7 hub genes through the cBioPortal database.

### Survival analysis and correlation analysis

The effects of the hub genes on overall survival (OS) and relapse-free survival (RFS) were determined by Kaplan Meier survival analysis. The log rank *P* value and hazard ratio (HR) with 95% confidence intervals are shown on the plot. Correlations between two hub genes were analyzed by the GEPIA online tool based on the TCGA database.

### Cell culture and transfection

The human HCC cell lines HepG2 and Huh7 and the normal hepatic cell line LO2 were purchased from the Type Culture Collection of the Chinese Academy of Sciences (Shanghai, China). All cell lines were maintained in Dulbecco’s modified Eagle medium (Gibco, US) supplemented with 10% fetal bovine serum (Gibco, US), 100 U/ml penicillin and 100 µg/ml streptomycin (Gibco, US). Cells were cultured at 37 °C in 5% CO2. Overexpression plasmid and small interfering RNAs (siRNA) targeting BUB1B were purchased from ShenGong (Shanghai, China). SiRNA and plasmid were transfected into HCC cells with Lipofectamine 2000 Reagent (Invitrogen, US) according to the manufacturer’s instructions. The siRNA sequences of BUB1B were as follows: siRNA#1 (si 1): 5′-GAGAGUAAUAUGUCAACGUUATT-3′, siRNA#2 (si 2): 5′-GCGUUUAUGCAAUGAGCCUUUTT-3′, and siRNA#3 (si 3): 5′-GAGACAACUAAACUGCAAAUUTT-3′.

### Colony formation assay

Suspended HCC cells transfected with BUB1B-siRNA or normal control were seeded into six-well plates (1,500 cells/well) in two mL of complete medium. The cells were cultured for 2 weeks. The colonies were stained with 0.5% crystal violet for 20 min, and the colony numbers were counted.

### Cell counting Kit-8 (CCK-8) assay

Cell proliferation and viability were assessed by CCK-8 assay (Dojindo, Japan) according to the manufacturer’s instructions. Briefly, HCC cells were seeded into 96-well plates at a density of 1,500 cells/well with 100 µl of culture medium. A total of 10 µl of CCK-8 reagent was added to each well at the indicated time points (24, 48 and 72 h). The plates were incubated in the dark at 37 °C for 2 h, and then the optical density was measured at 450 nm. The experiments were performed in triplicate.

### Migration and invasion assays

Cell migration and invasion assays were carried out in Transwell chambers (Corning, US). For the cell migration assay, a total of 4 × 104 cells in 200 µl of serum-free DMEM were seeded into the upper chamber. For the invasion assay, 4 × 104 cells suspended in serum-free medium were plated in the upper chamber coated with Matrigel (BD Biosciences, US). Lower chambers were filled with 500 µl of complete medium, and the cells were cultured for 48 h. The cells that passed through the membrane were stained with 0.5% crystal violet for 15 min. Cells were counted in five random areas to evaluate the migration and invasion abilities. The experiments were performed in triplicate.

### Quantitative real-time PCR (qRT-PCR)

Total RNA was extracted from HCC cells and normal hepatic cells using TRIzol reagent (Invitrogen, US). The procedure was performed as described previously (Fang et al., 2018). GAPDH was used as an internal control. Relative expression levels of BUB1B were calculated according to the 2 ΔΔCt method. All of the primers in this study were synthesized by ShenGong (Shanghai, China). The BUB1B primer sequences were as follows: (forward) 5′-CTGAGTGAAGCCATGTCCCT-3′ and (reverse) 5′-AGATTCTTGTGCCAGTGCTCCC-3′. The GAPDH primer sequences were as follows: (forward) 5′-GCGACTTCAACAGCAACT CCC-3′ and (reverse) 5′-CACCCTGTTGCTGTAGCCGTA-3′.

### Flow cytometry

The cell apoptosis rate and cell cycle were measured by flow cytometry. Briefly, HCC cells were subjected to various treatments, stained with 5 µl of FITC Annexin V and 5 µl PI (BD, US) for 15 min at RT in the dark, then cell apoptosis rate were analyzed by flow cytometry. For cell cycle, HCC cells after treatments were fixed in 70% ethanol at −20 °C overnight. On the next day, cells were stained with PI (50 µg/mL, Sigma-Aldrich, US) for 20 min at room temperature in the dark and analyzed by flow cytometry.

### ATP and mitochondrial membrane potential detection

Total ATP levels in HCC cells were measured using a luciferase-based ATP assay kit (Beyotime, China) according to the manufacturer’s instructions. The mitochondrial membrane potential was analyzed using JC 1 staining (Beyotime, China). Briefly, cells were washed with ice-cold PBS and then stained with JC-1 for 20 min at 37 °C. After being washed with binding buffer, the cells were analyzed by fluorescence microscopy. The results are presented as the relative aggregate-to-monomer (red/green) fluorescence intensity ratio.

### Oxygen consumption rate (OCR) analysis

Mitochondrial OCR of HCC cells were measured using the XF 24 analyzer (Seahorse Bioscience, US) according to the manufacturer’s instructions as previously described (Meng et al., 2017). Briefly, HepG2 and Huh7 cells treated with BUB1B siRNA or normal control were seeded into XF-24 microplates at 37 °C with 5% CO2. Cells were maintained at 37 °C in a non CO2 incubator for 1 h, and then the basal OCR of the HCC cells were measured. The OCR was normalized to the protein content.

### Statistical analysis

SPSS version 19.0 was used to analyze the data. All data were expressed as the mean ± SEM. Student’s *t*-test was used in the two-group comparisons, and one-way ANOVA was used for more than two groups. *P* value < 0.05 was considered statistically significant.

## Results

### Identification of DEGs in HCC

**Figure 1:**
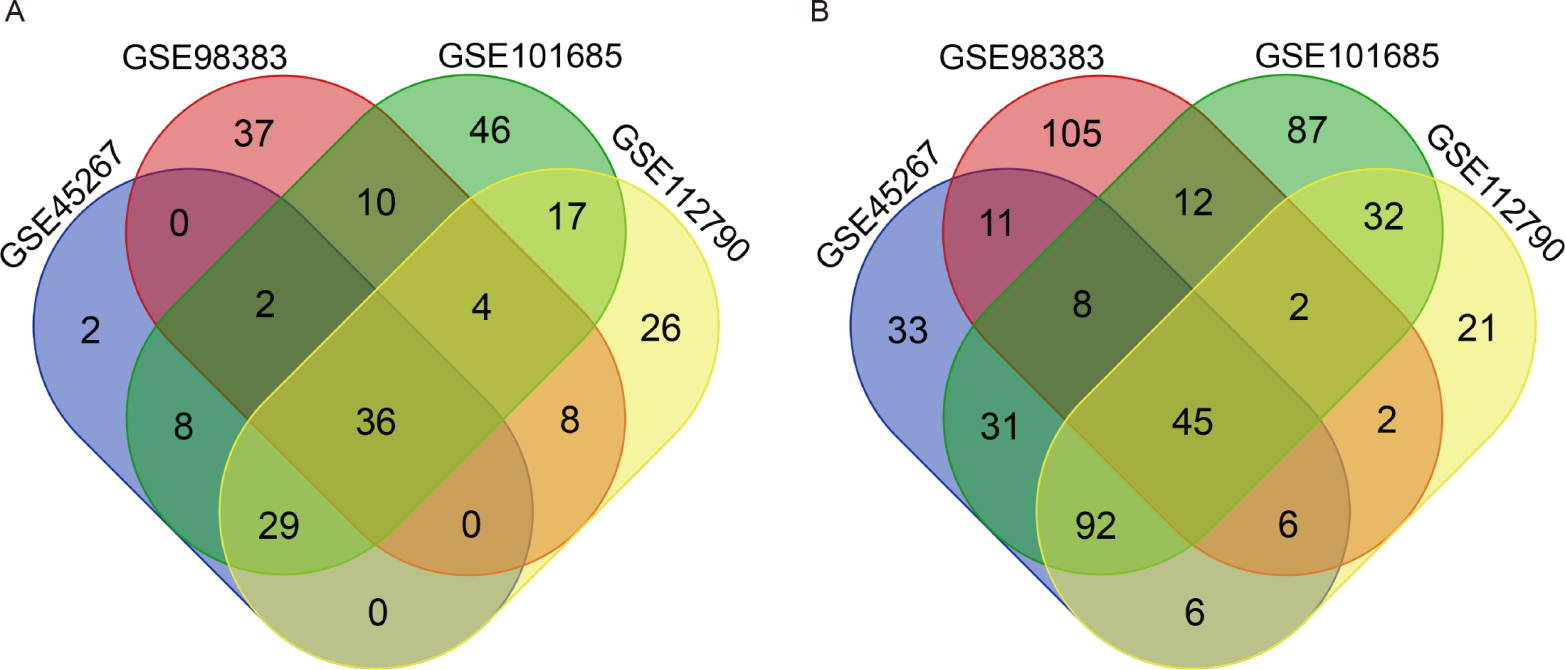
Identification of common DEGs from the GSE45267, GSE98383, GSE101685 and GSE112790 datasets. Venn diagram of (A) upregulated and (B) downregulated DEGs between HCC tissues and normal hepatic tissues based on the four GEO datasets. The intersecting areas represent the commonly altered DEGs. DEGs, differentially expressed genes; HCC, hepatocellular carcinoma; GEO, Gene Expression Omnibus.

There were 264 HCC tissues and 91 normal hepatic tissues included in our study. Via the GEO2R online tool, we identified 309, 288, 461 and 326 DEGs from GSE45267, GSE98383, GSE101685 and GSE112790, respectively. Then, we used a Venn diagram to identify the intersected DEGs among the four datasets. A total of 81 common DEGs were identified, of which 36 were upregulated (Fig. 1A) and 45 were downregulated (Fig. 1B) in HCC tissues compared with normal hepatic tissues (Table 1).

**Table 1:** Eighty-one common differentially expressed genes (DEGs) were detected from four GEO datasets, including 36 upregulated genes and 45 downregulated genes in the HCC tissues compared to normal hepatic tissues.

DEGs	Genes Name
Up-regulated	KIF20A CDKN3 IGF2BP3 RRM2 AKR1B10 CCNB1 CDK1 PBK NUF2 NDC80 COCH NCAPG DUXAP10 TOP2A CENPF GINS1 SPINK1 GPC3 CD109 MELK ASPM BUB1B MAD2L1 CENPU HMMR DLGAP5 KIF4A DTL CCNB2 TTK CDC20 FAM83D NEK2 ANLN PRC1 BIRC5
Down-regulated	CETP CRHBP LPA RSPO3 EGR1 ADRA1A CLEC1B IGFBP3 FOS FCN3 FOSB HHIP LIFR APOF APOA5 HAMP PDGFRA DNASE1L3 DCN TTC36 PGLYRP2 ADH1B CLEC4G C9 LINC01093 MARCO ADH4 CXCL14 GPM6A IGF1 SRPX SLCO1B3 CYP2E1 NNMT OIT3 HGF ADGRG7 LCAT CXCL12 GNMT C7 FCN2 LYVE1 SLC25A47 MFSD2A

### GO annotation and KEGG pathway analysis of DEGs

GO annotation and KEGG pathway enrichment analysis of all 81 DEGs were performed by the DAVID online tool. The top 5 enriched GO terms are listed (Table 2). The results of the GO BP analysis indicated that the upregulated DEGs were enriched in mitotic nuclear division, cell division, sister chromatid cohesion, G2/M transition of mitotic cell cycle, and mitotic spindle assembly checkpoint. The downregulated DEGs were enriched in lipoprotein metabolic process, phosphatidylcholine metabolic process, cell chemotaxis, positive regulation of phosphatidylinositol 3-kinase signaling, and lipid transport. In the GO CC analysis, upregulated DEGs were significantly enriched in the midbody, condensed chromosome kinetochore, kinetochore, spindle pole, and spindle. In addition, downregulated DEGs were enriched in extracellular region, high-density lipoprotein particle, extracellular space, integral component of plasma membrane, and blood microparticle. In the GO MF analysis, upregulated DEGs were significantly enriched in protein binding, microtubule binding, protein serine/threonine kinase activity, cyclin-dependent protein serine/threonine kinase activity, and protein kinase activity. The downregulated DEGs were enriched in cholesterol binding, zinc-dependent alcohol dehydrogenase activity, serine-type endopeptidase activity, phospholipid transporter activity, cholesterol transporter activity. In addition, the top 4 enriched KEGG pathways of DEGs were listed as Table 3. Upregulated genes were enriched in cell cycle, oocyte meiosis, p53 signaling pathway, progesterone-mediated oocyte maturation. And downregulated genes were enriched in prion diseases, pathways in cancer, drug metabolism-cytochrome P450, and melanoma.

**Table 2:** Gene Ontology analysis of DEGs in HCC.

Expression	Category	Term	Count	*p*-Value	FDR
Up-	GOTERM_BP_DIRECT	GO:0007067∼mitotic nuclear division	13	3.39E−14	4.51E−11
regulated	GOTERM_BP_DIRECT	GO:0051301∼cell division	13	2.02E−12	2.70E−09
	GOTERM_BP_DIRECT	GO:0007062∼sister chromatid cohesion	8	1.25E−09	1.66E−06
	GOTERM_BP_DIRECT	GO:0000086∼G2/M transition of mitotic cell cycle	7	2.94E−07	3.93E−04
	GOTERM_BP_DIRECT	GO:0007094∼mitotic spindle assembly checkpoint	4	8.44E−06	0.011255
	GOTERM_CC_DIRECT	GO:0030496∼midbody	8	3.47E−09	3.69E−06
	GOTERM_CC_DIRECT	GO:0000777∼condensed chromosome kinetochore	7	1.20E−08	1.28E−05
	GOTERM_CC_DIRECT	GO:0000776∼kinetochore	6	3.85E−07	4.09E−04
	GOTERM_CC_DIRECT	GO:0000922∼spindle pole	6	1.69E−06	0.001798
	GOTERM_CC_DIRECT	GO:0005819∼spindle	6	2.83E−06	0.003011
	GOTERM_MF_DIRECT	GO:0005515∼protein binding	31	8.61E−06	0.009201
	GOTERM_MF_DIRECT	GO:0008017∼microtubule binding	5	7.77E−04	0.827275
	GOTERM_MF_DIRECT	GO:0004674∼protein serine/threonine kinase activity	6	8.73E−04	0.928326
	GOTERM_MF_DIRECT	GO:0004693∼cyclin-dependent protein serine/threonine kinase activity	3	0.002121	2.24328
	GOTERM_MF_DIRECT	GO:0004672∼protein kinase activity	5	0.005637	5.860713
Down-	GOTERM_BP_DIRECT	GO:0042157∼lipoprotein metabolic process	4	1.15E−04	0.16458
regulated	GOTERM_BP_DIRECT	GO:0046470∼phosphatidylcholine metabolic process	3	2.71E−04	0.386322
	GOTERM_BP_DIRECT	GO:0060326∼cell chemotaxis	4	5.71E−04	0.810655
	GOTERM_BP_DIRECT	GO:0014068∼positive regulation of phosphatidylinositol 3-kinase signaling	4	5.71E−04	0.810655
	GOTERM_BP_DIRECT	GO:0006869∼lipid transport	4	9.01E−04	1.277227
	GOTERM_CC_DIRECT	GO:0005576∼extracellular region	19	4.40E−09	4.54E−06
	GOTERM_CC_DIRECT	GO:0034364∼high-density lipoprotein particle	4	1.70E−05	0.017543
	GOTERM_CC_DIRECT	GO:0005615∼extracellular space	11	7.78E−04	0.80017
	GOTERM_CC_DIRECT	GO:0005887∼integral component of plasma membrane	10	0.004284	4.333163
	GOTERM_CC_DIRECT	GO:0072562∼blood microparticle	4	0.005147	5.185309
	GOTERM_MF_DIRECT	GO:0015485∼cholesterol binding	3	0.004029	4.881137
	GOTERM_MF_DIRECT	GO:0004024∼alcohol dehydrogenase activity, zinc-dependent	2	0.013784	15.80658
	GOTERM_MF_DIRECT	GO:0004252∼serine-type endopeptidase activity	4	0.020868	23.00373
	GOTERM_MF_DIRECT	GO:0005548∼phospholipid transporter activity	2	0.02287	24.93304
	GOTERM_MF_DIRECT	GO:0017127∼cholesterol transporter activity	2	0.034113	34.96525

**Table 3:** KEGG pathway analysis of DEGs in HCC.

Expression	Term	Count	*p*-Value	Genes
Upregulated	hsa04110: Cell cycle	7	1.30E−08	CCNB1, CDK1, MAD2L1, CCNB2, TTK, BUB1B, CDC20
	hsa04114: Oocyte meiosis	5	1.94E−05	CCNB1, CDK1, MAD2L1, CCNB2, CDC20
	hsa04115: p53 signaling pathway	4	1.38E−04	CCNB1, CDK1, CCNB2, RRM2
	hsa04914: Progesterone-mediated oocyte maturation	4	3.00E−04	CCNB1, CDK1, MAD2L1, CCNB2
Downregulated	hsa05020: Prion diseases	3	0.005622	EGR1, C7, C9
	hsa05200: Pathways in cancer	6	0.008486	FOS, PDGFRA, IGF1, HHIP, HGF, CXCL12
	hsa00982: Drug metabolism- cytochrome P450	3	0.02131	ADH4, ADH1B, CYP2E1
	hsa05218: Melanoma	3	0.023107	PDGFRA, IGF1, HGF

### PPI and modular analysis

**Figure 2:**
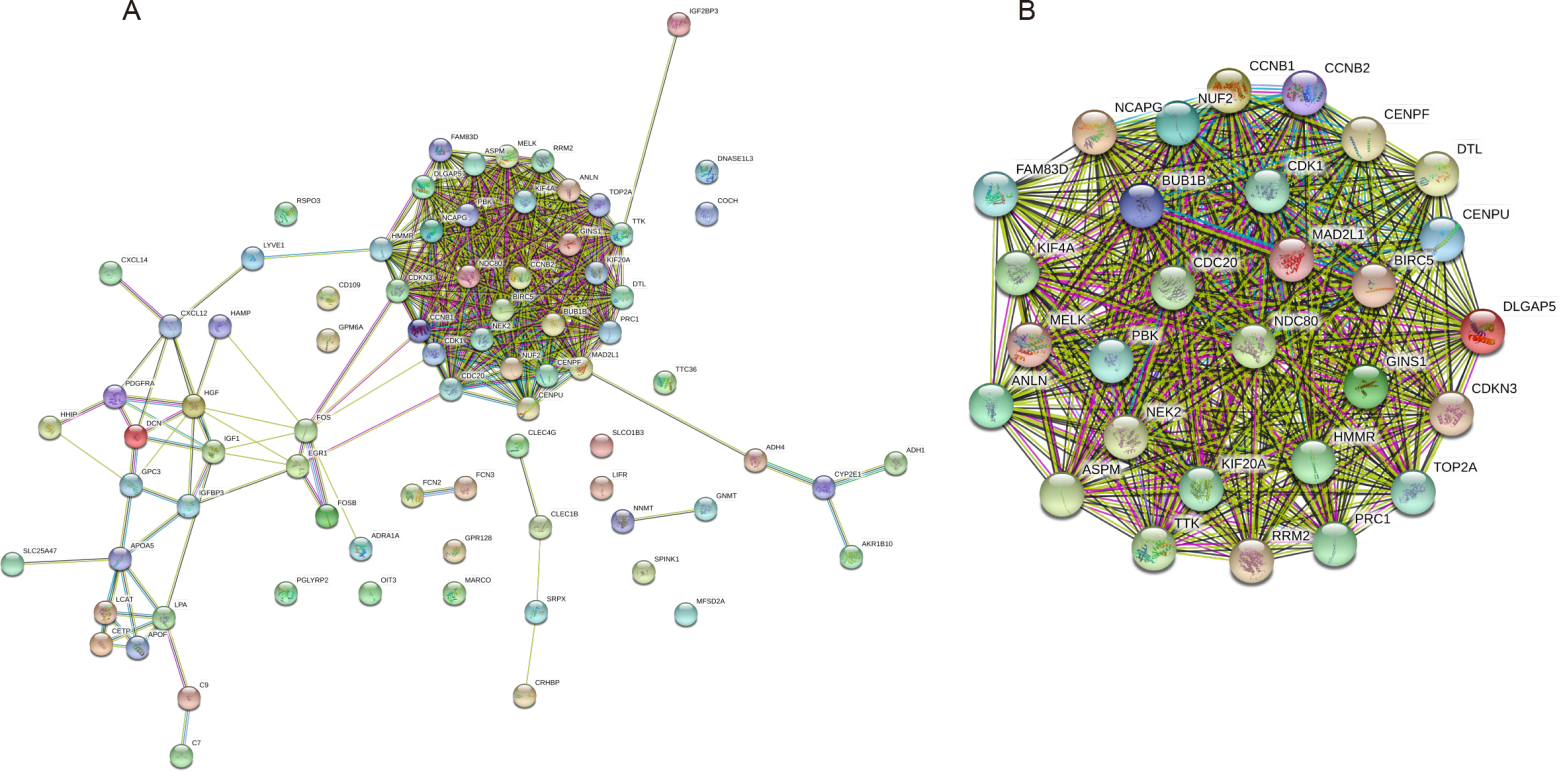
PPI network construction and module analysis. (A) A PPI network was constructed based on all 81 DEGs using the STRING database. Module analysis of the PPI network was performed by the MCODE plugin of Cytoscape. (B) Twenty-nine central nodes were identified. PPI, protein–protein interaction; DEGs, differentially expressed genes; STRING, Search Tool for the Retrieval of Interacting Genes.

The PPI network of the 81 DEGs was constructed by the STRING database, as shown in Fig. 2A. Then, we used the MCODE plugin of Cytoscape software for further modular analysis. The results showed that 29 central nodes were identified (Fig. 2B). Further KEGG analysis of the 29 genes in the central nodes showed that they were significantly enriched in the cell cycle, oocyte meiosis, p53 signaling pathway, progesterone-mediated oocyte maturation and HTLV-I infection (Table 4). The remaining 7 genes found in significantly enriched pathways, namely, mitotic checkpoint serine/threonine kinase B (BUB1B), cyclin B1 (CCNB1), cyclin B2 (CCNB2), cell division cycle 20 (CDC20), cyclin-dependent kinase 1 (CDK1), MAD2 mitotic arrest deficient-like 1 (MAD2L1) and ribonucleotide reductase M2 (RRM2), were regarded as hub genes.

### Validation of the expression levels of the 7 Hub genes in HCC

**Figure 3:**
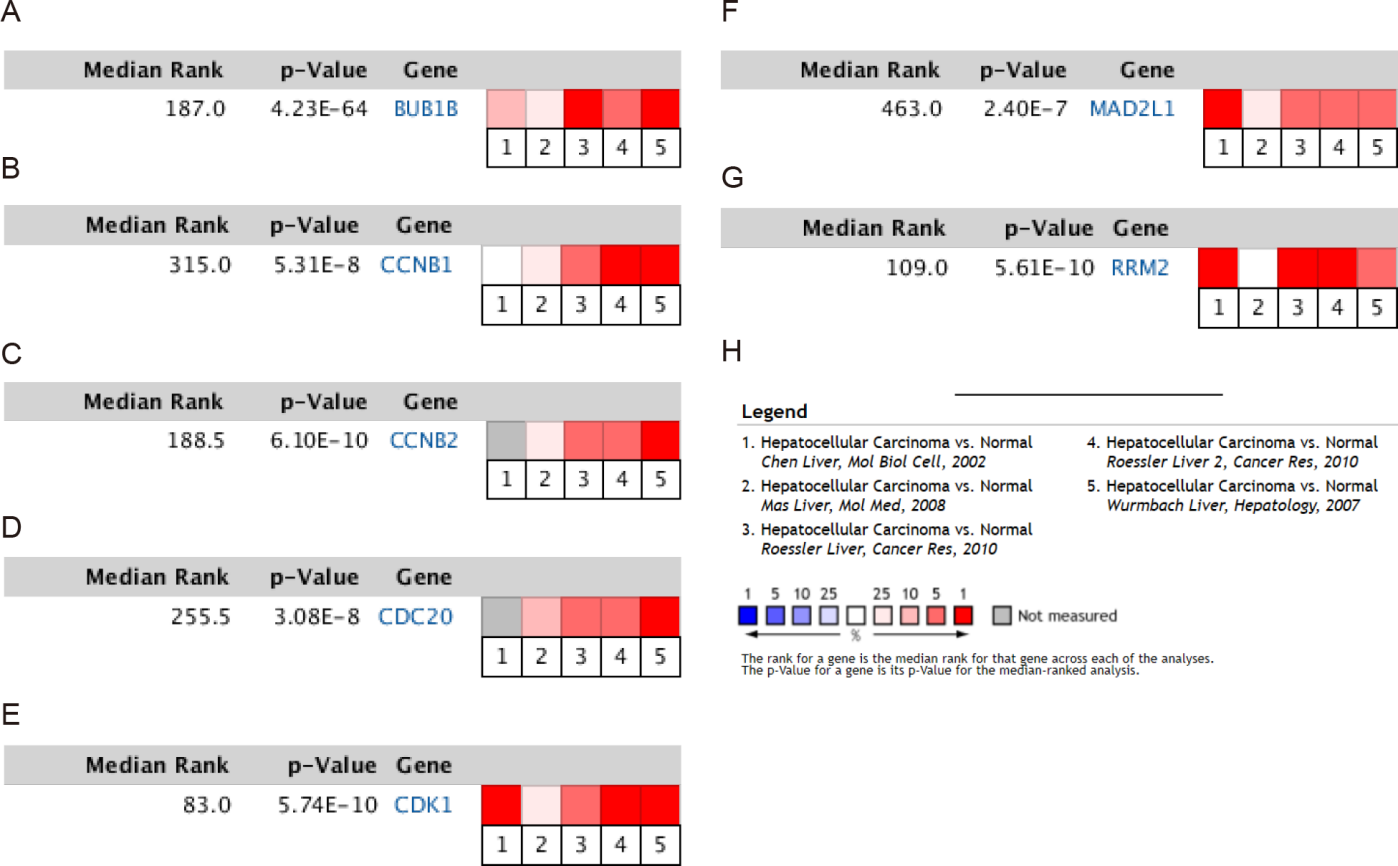
Meta-analysis of the mRNA expression levels of (A) BUB1B, (B) CCNB1, (C) CCNB2, (D) CDC20, (E) CDK1, (F) MAD2L1 and (G) RRM2 in HCC tissues compared with normal hepatic tissues using the ONCOMINE database. The colored squares represent the median rank of these genes across five datasets in ONCOMINE. *P* < 0.05 was regarded as statistically significant. HCC, hepatocellular carcinoma. The expression level is described by *Z*-score.

**Figure 4:**
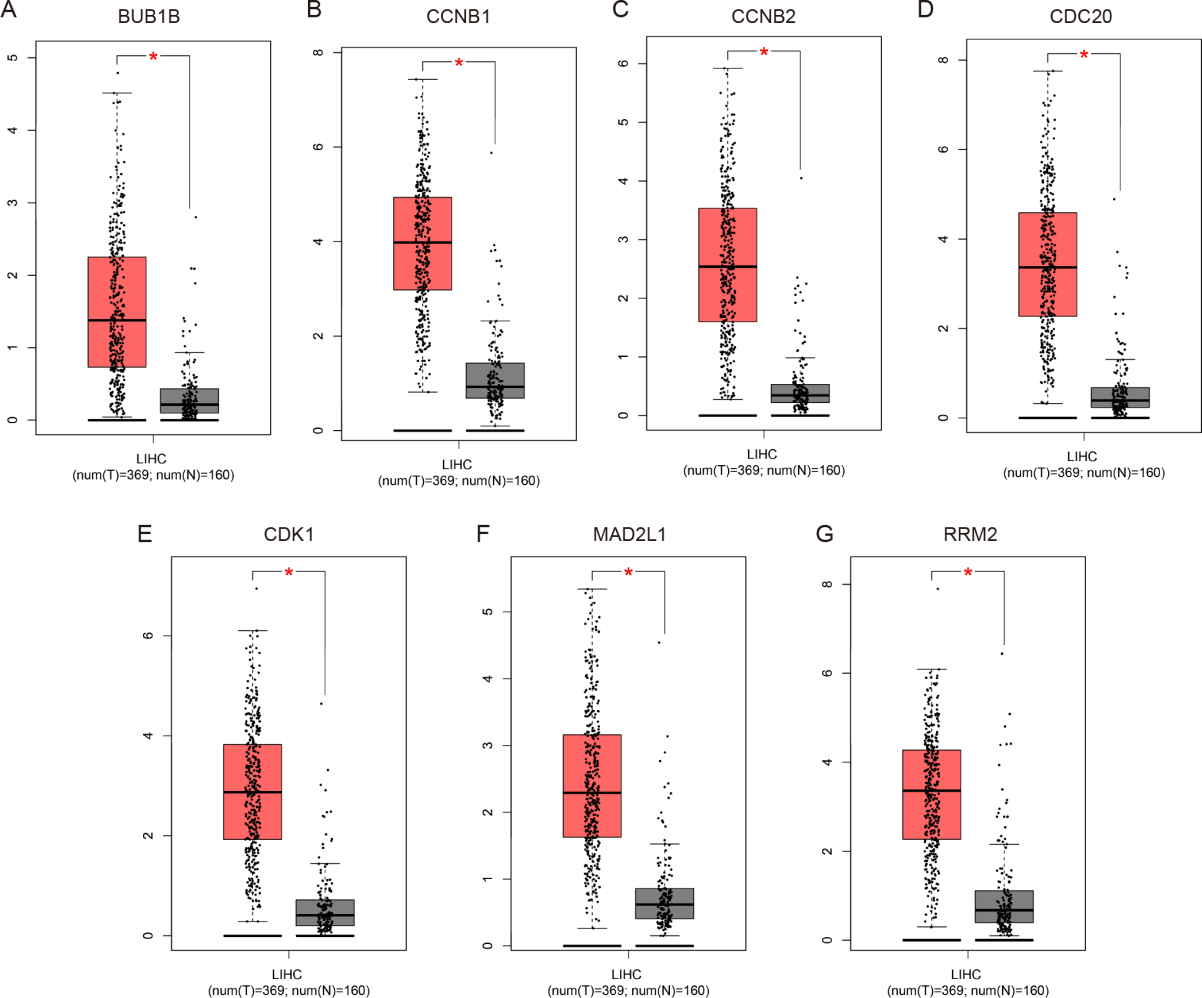
Validation of the mRNA expression levels of (A) BUB1B, (B) CCNB1, (C) CCNB2, (D) CDC20, (E) CDK1, (F) MAD2L1 and (G) RRM2 in LIHC tissues compared with normal hepatic tissues using the GEPIA online tool. These box plots are based on 369 hepatocellular carcinoma samples (red) and 160 normal liver samples (gray). *P < 0.05 was considered statistically significant. LIHC, liver hepatocellular carcinoma. The expression level is described by log2(TPM + 1).

To validate the mRNA expression levels of the screened hub genes between HCC and normal hepatic tissues, a meta-analysis on the mRNA expression levels of BUB1B, CCNB1, CCNB2, CDC20, CDK1, MAD2L1 and RRM2 was performed based on the ONCOMINE database. As displayed in Fig. 3, all the hub genes were significantly highly expressed in HCC tissues (*P* < 0.05) compared with normal hepatic tissues. The results from the GEPIA database also revealed that the mRNA levels of all 7 hub genes were significantly highly expressed in HCC tissues (Fig. 4). These results were consistent with previous microarray data.

**Table 4:** Reanalysis of the 29 genes in the central nodes by KEGG pathway enrichment.

Term	Count	p-Value	Genes
hsa04110: Cell cycle	6	9.42E−08	CCNB1, CDK1, MAD2L1, CCNB2, BUB1B, CDC20
hsa04114: Oocyte meiosis	5	4.28E−06	CCNB1, CDK1, MAD2L1, CCNB2, CDC20
hsa04115: p53 signaling pathway	4	4.78E−05	CCNB1, CDK1, CCNB2, RRM2
hsa04914: Progesterone-mediated oocyte maturation	4	1.05E−04	CCNB1, CDK1, MAD2L1, CCNB2
hsa05166: HTLV-I infection	3	0.032823	MAD2L1, BUB1B, CDC20

### Genetic alterations and prognostic values of the hub genes

**Figure 5:**
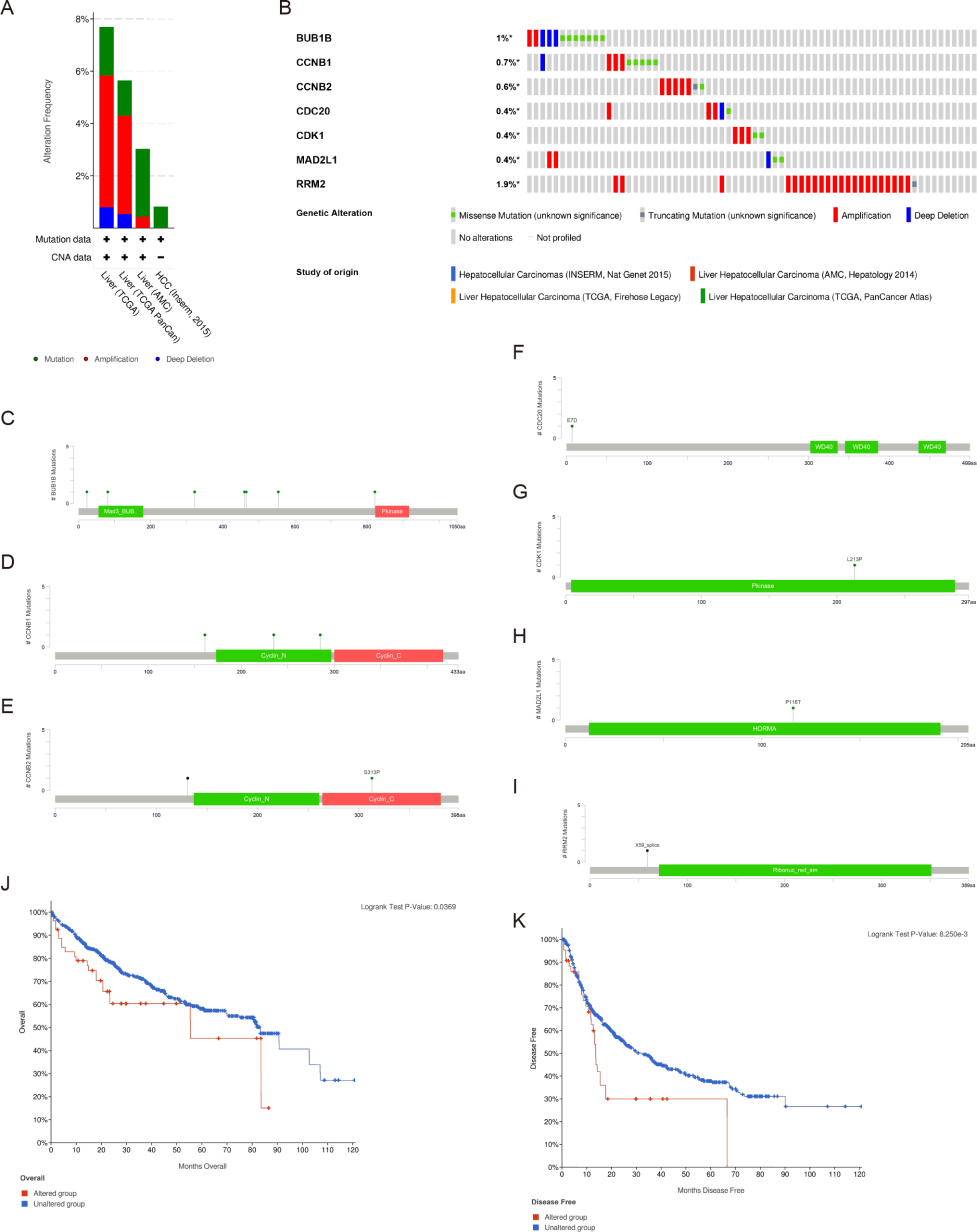
Genetic alterations and prognostic values of the seven hub genes. (A, B) The frequencies of genetic alterations of the seven hub genes in HCC tissues were identified by cBioPortal. The specific mutation information for (C) BUB1B, (D) CCNB1, (E) CCNB2, (F) CDC20, (G) CDK1, (H) MAD2L1 and (I) RRM2 is displayed individually. (J, K) HCC cases with altered hub gene expression exhibited significantly worse OS and DFS compared to those with unaltered hub gene expression. HCC, hepatocellular carcinoma. OS, overall survival; DFS, disease-free survival.

There were significant genetic alterations in the 7 hub genes in the HCC cases, as evaluated by the online tool cBioPortal. The mRNA sequences of the 7 hub genes in HCC were amplified (Figs. 5A, 5B). The specific mutation information of each hub gene is displayed individually (Figs. 5C–5I). Kaplan Meier plots were used to compare OS and DFS in HCC patients with or without alterations in the mRNA expression levels of the 7 hub genes through cBioPortal. As revealed in Figs. 5J, 5K, HCC cases with altered hub gene expression exhibited significantly worse OS and DFS than those with unaltered hub gene expression.

### Survival analysis of the hub genes

**Figure 6:**
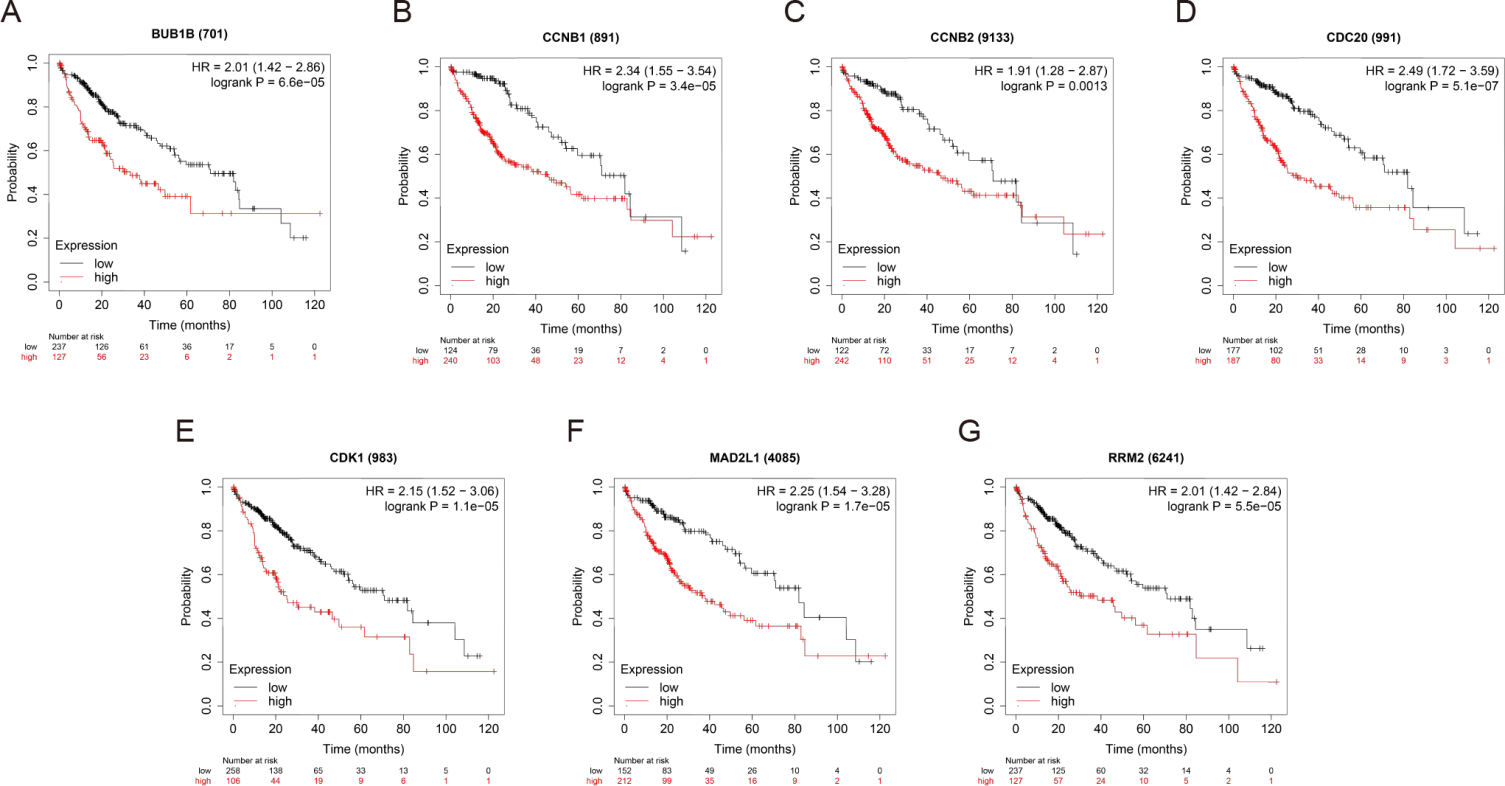
OS of the seven hub genes (A) BUB1B, (B) CCNB1, (C) CCNB2, (D) CDC20, (E) CDK1, (F) MAD2L1 and (G) RRM2 in patients with HCC were analyzed by Kaplan–Meier plotter. The data are presented as the hazard ratios with 95% confidence intervals. Log–rank *P* < 0.01 was regarded as statistically significant. OS, overall survival; HCC, hepatocellular carcinoma.

**Figure 7:**
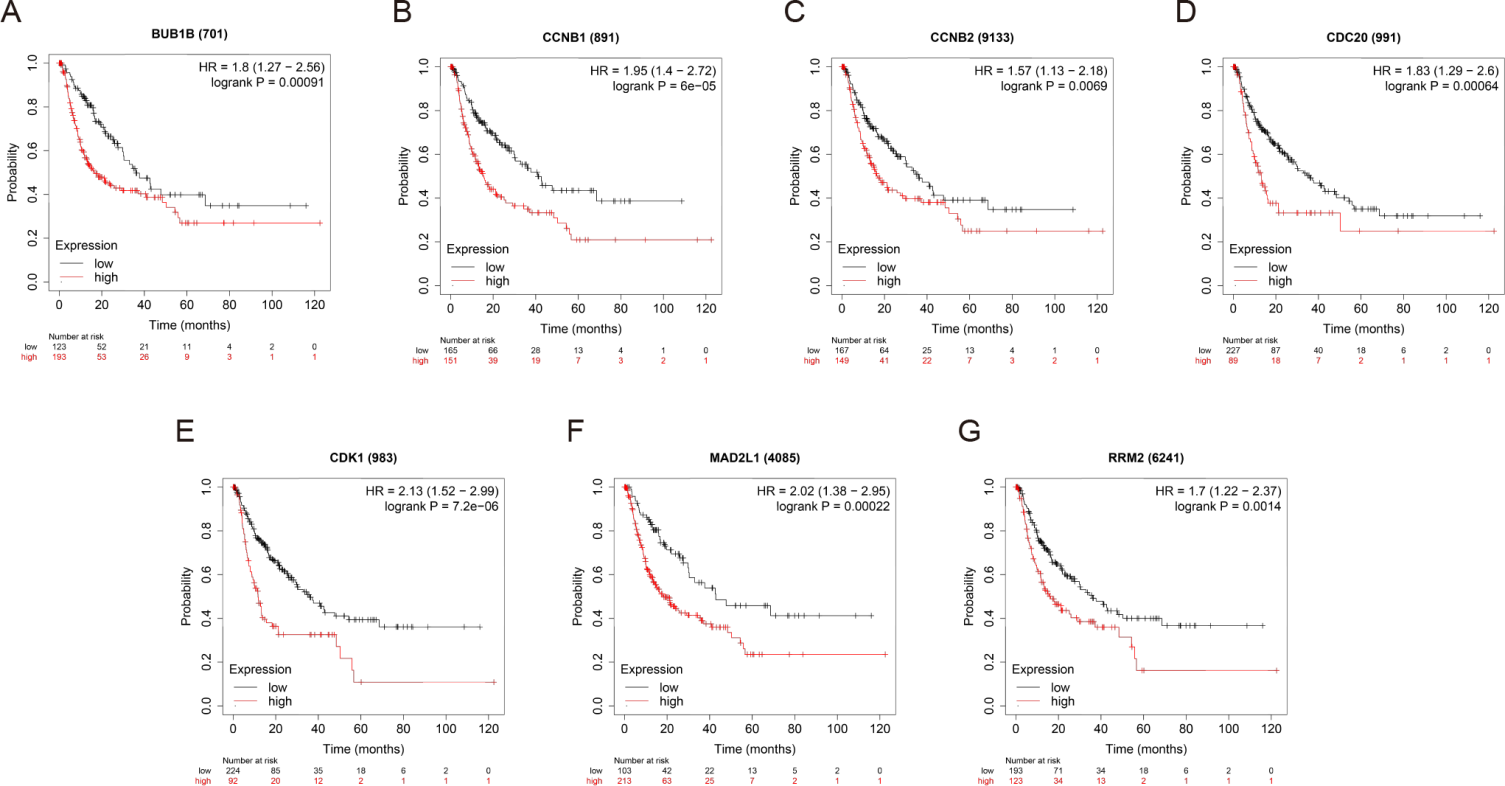
RFS of the seven hub genes (A) BUB1B, (B) CCNB1, (C) CCNB2, (D) CDC20, (E) CDK1, (F) MAD2L1 and (G) RRM2 in patients with HCC were analyzed by Kaplan–Meier plotter. The data are presented as hazard ratios with 95% confidence intervals. Log–rank *P* < 0.01 was regarded as statistically significant. RFS, relapse-free survival; HCC, hepatocellular carcinoma.

OS and RFS analyses of the 7 hub genes were further performed by Kaplan Meier plotter. As displayed in Fig. 6, the high expression levels of BUB1B, CCNB1, CCNB2, CDC20, CDK1, MAD2L1 and RRM2 in patients with HCC were associated with poor OS. Unfavorable RFS was also markedly observed in HCC patients with higher expression levels of the 7 hub genes (Fig. 7). Patients were divided into two groups (“high” and “low” group) according to 50% cut-off value.

### Decreased BUB1B expression inhibited the proliferation, migration, and invasion, promoted the apoptosis and blocked cell cycle of hcc cells

**Figure 8:**
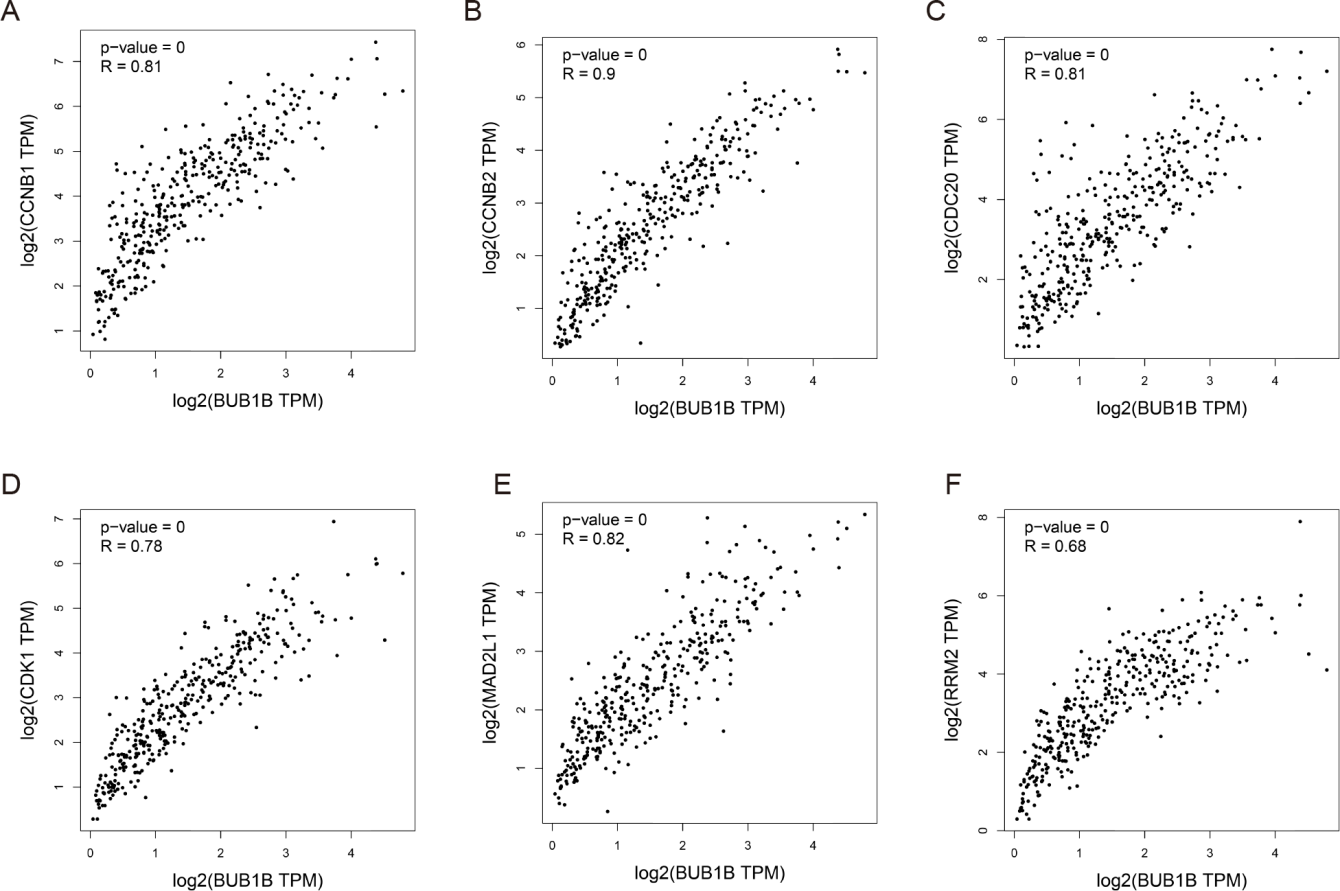
Correlations between BUB1B and (A) CCNB1, (B) CCNB2, (C) CDC20, (D) CDK1, (E) MAD2L1 and (F) RRM2 were analyzed by GEPIA. GEPIA, gene expression profiling interactive analysis.

**Figure 9:**
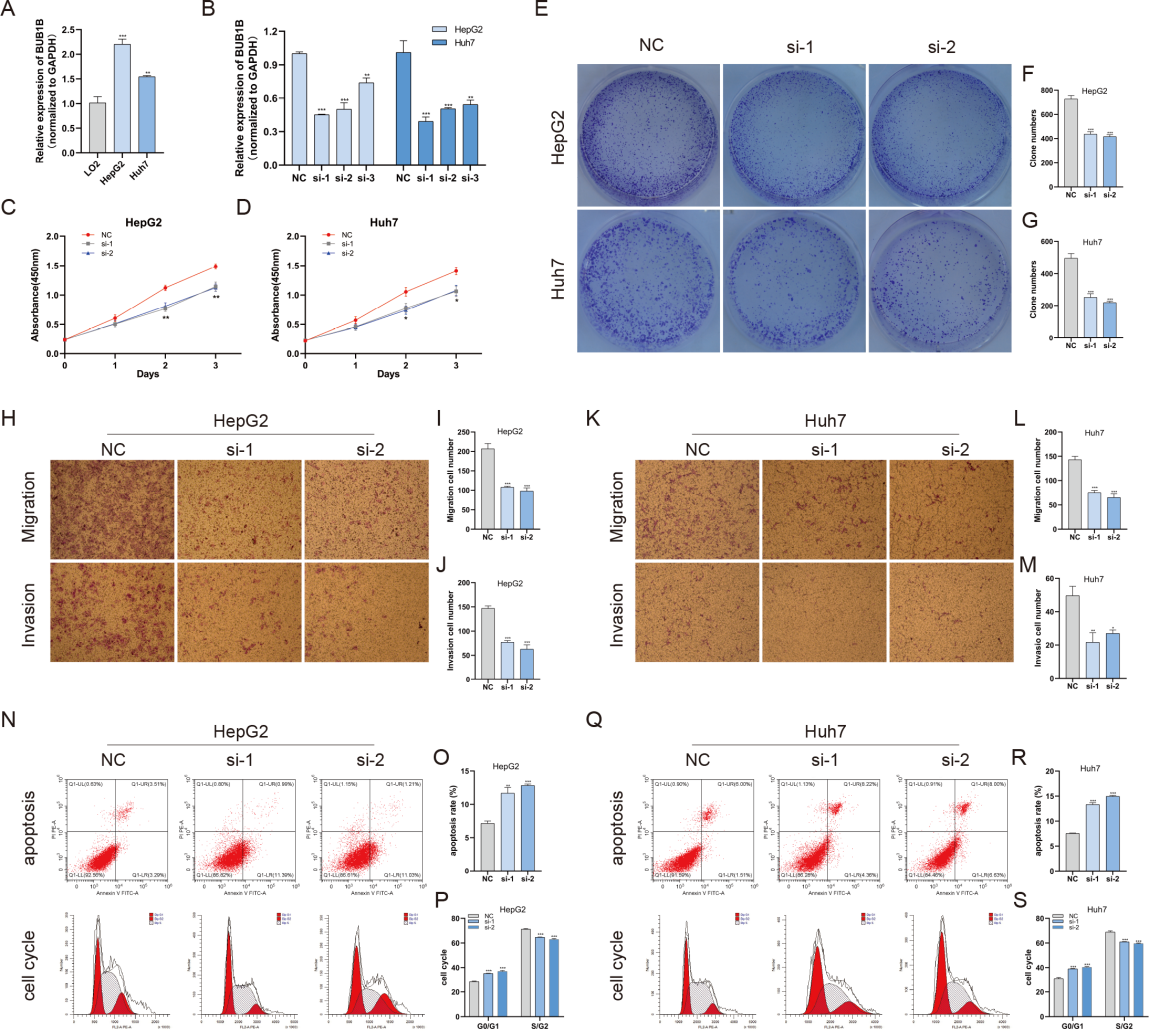
Decreased BUB1B expression inhibited the proliferation, migration, and invasion, promoted the apoptosis and blocked cell cycle of HCC cells. (A) The mRNA expression levels of BUB1B were detected in HCC cells and normal hepatic cells. (B) qRT-PCR was used to confirm the knockdown efficiency of the siRNAs against BUB1B. (C–G) CCK-8 assays and colony formation assays were performed to evaluate the proliferation of the HCC cell lines. (H–M) The effects of BUB1B knockdown on cell migration and invasion were determined by Transwell assays. (N–S) The cell apoptosis rate and cell cycle were analyzed by flow cytometry in HCC cells. HCC, hepatocellular carcinoma; CCK-8, Cell Counting Cit-8. ^∗^*P* < 0.05, ^∗∗^*P* < 0.01, ^∗∗∗^*P* < 0.001.

Except for BUB1B, the mechanisms of the other hub genes in hepatocarcinogenesis have been elucidated to varying degrees (Foijer et al., 2017; Jin et al., 2020; Li et al., 2014; Li, Bai & Zhang, 2017; Liu et al., 2020; Wu et al., 2018b; Yang, Lin & Liu, 2020). Therefore, the relationship between BUB1B and other hub genes was investigated first. As shown in Fig. 8, BUB1B was significantly associated with the other six hub genes. To further investigate the specific biological role of BUB1B in HCC, in vitro mechanistic experiments were conducted. The expression levels of BUB1B in HCC cells and normal hepatic cells were detected by qRT-PCR. The results showed that BUB1B mRNA levels were obviously higher in the HCC cell lines HepG2 and Huh7 than in the normal cell line LO2 (Fig. 9A). To assess the biological functions of BUB1B in HCC, we transfected small interfering RNA (siRNA) or negative control into HepG2 and Huh7 cell lines. The knockdown efficiency of BUB1B by the siRNAs was confirmed using qRT-PCR (Fig. 9B). The results suggested that si-1 and si-2 had clear knockdown effects and were selected for subsequent functional studies. CCK-8 assays confirmed the inhibitory effect of decreased BUB1B expression on HCC cells (Fig. 9C). Moreover, colony formation assays indicated that BUB1B downregulation significantly suppressed the proliferation of HepG2 and Huh7 cells (Fig. 9D). Next, the effects of BUB1B on the migration and invasion of HCC cells were explored by Transwell assays. The results demonstrated that the numbers of migrated and invaded cells were significantly lower in the BUB1B knockdown group than in the control group (Figs. 9E, 9F). We further investigated whether BUB1B played a role in the apoptosis and cell cycle of HCC cells. Flow cytometry indicated that BUB1B knockdown resulted in a significantly higher apoptosis rate and G0/G1 phase arrest compared with that in the control group (Figs. 9G, 9H). These results indicate that downregulated BUB1B significantly inhibited the proliferation, migration, and invasion, promoted the apoptosis and blocked cell cycle of HCC cells in vitro. The oncogenic role of BUB1B in HCC was further validated by its overexpression (Supporting Fig. 1).

### BUB1B plays a role in mitochondrial function

**Figure 10:**
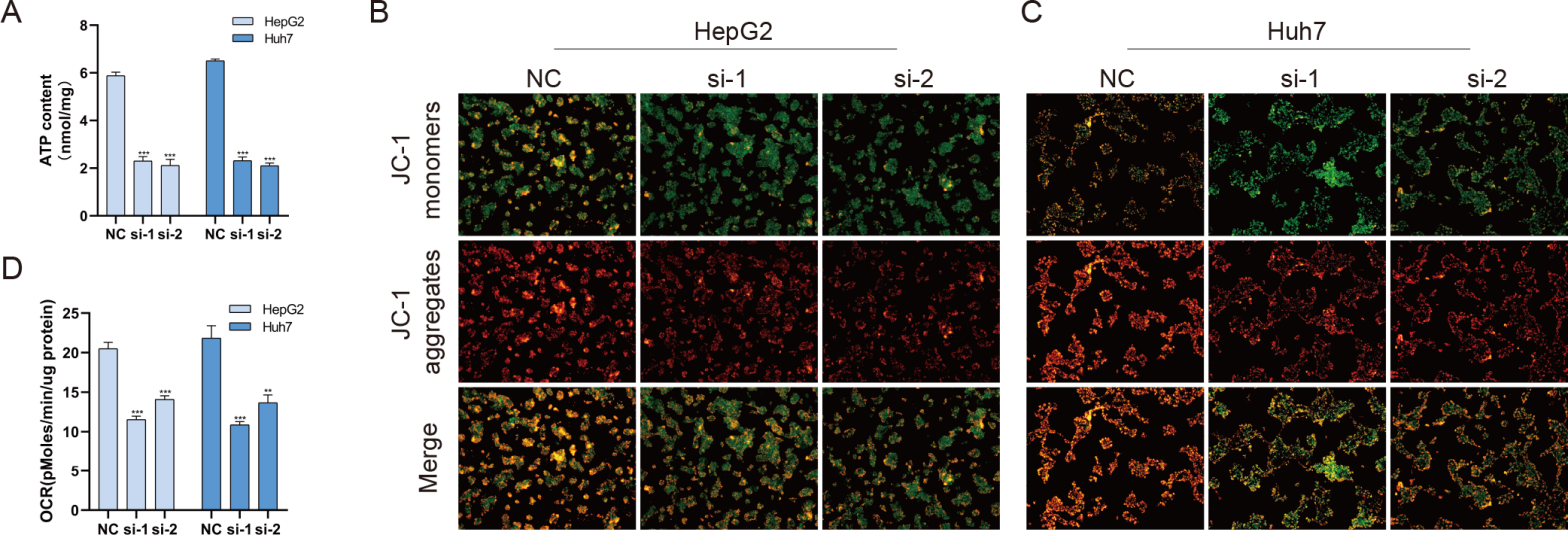
BUB1B plays a role in mitochondrial function. Total ATP production was detected in different treatment groups (A). Mitochondrial membrane potential was analyzed by JC-1 staining (B, C). The basal OCR of HCC cells were measured using an XF-24 analyzer (D). OCR, oxygen consumption rate; HCC, hepatocellular carcinoma. ^∗^*P* < 0.05, ^∗∗^*P* < 0.01, ^∗∗∗^*P* < 0.001.

Mitochondria are involved in energy synthesis, apoptosis and other important biological processes and are vital for cell survival and function. Recently, mitochondrial dysfunction has been reported to participate in tumorigenesis (Davis et al., 2020; Vyas, Zaganjor & Haigis, 2016; Zhang et al., 2019). To further understand the mechanistic oncogenic role of BUB1B, mitochondrial bioenergetics were investigated. As shown in Fig. 10A, total ATP production was significantly downregulated after BUB1B knockdown. JC-1 staining demonstrated that the mitochondrial membrane potential, which is a biomarker of apoptosis, was also reduced after BUB1B deletion (Fig. 10B). Next, the mitochondrial OCR of HCC cells were measured using the XF-24 analyzer. As shown in Fig. 10C, the basal OCR was significantly decreased after BUB1B knockdown. On the contrary, overexpression of BUB1B increased OCR, ATP content and mitochondrial membrane potential (Supporting Fig. 2). These results indicate that BUB1B exerts its oncogenic effect partially by affecting mitochondrial function.

## Discussion

To identify more specific and effective biomarkers in HCC that are closely related to tumorigenesis, chemoresistance and prognosis, a combination analysis of bioinformatic methods and chip data based on a large number of clinical samples was conducted. In this study, four GEO datasets (GSE45267, GSE98383, GSE101685 and GSE112790) were analyzed. DEGs between HCC tissues and normal hepatic tissues were identified by GEO2R. Next, PPI network, GO annotation and KEGG pathway enrichment analyses of the DEGs were conducted. There were 29 central nodes identified by STRING and the MCODE plugin of Cytoscape. Further KEGG analysis of these 29 genes showed that they were significantly enriched in the cell cycle, oocyte meiosis, the p53 signaling pathway, progesterone-mediated oocyte maturation and HTLV-I infection. The remaining 7 genes found in significantly enriched pathways, namely, BUB1B, CCNB1, CCNB2, CDC20, CDK1, MAD2L1 and RRM2, were regarded as hub genes. The mRNA expression levels of the 7 hub genes in HCC were further validated based on the online databases ONCOMINE and GEPIA. Consistent with previous results, the 7 hub genes were significantly upregulated in HCC tissues. Next, the frequencies of the genetic alterations and prognostic roles of the 7 hub genes were evaluated by the online tool cBioPortal. Finally, the results showed unfavorable OS and RFS in HCC patients with higher expression levels of the 7 hub genes, which were confirmed by Kaplan Meier plotter.

CCNB1, CCNB2, CDK1 and CDC20, are all cell cycle-related proteins. Many studies have demonstrated their oncogenic roles in the regulation of cell cycle progression in a series of tumors, such as hepatocellular carcinoma (Jin et al., 2020; Li et al., 2014; Liu et al., 2020; Wu et al., 2018a), bladder cancer (Heo et al., 2020; Kim et al., 2014), non-small lung cancer (Qian et al., 2015; Wang et al., 2019a), pancreatic cancer (Wei et al., 2013; Zhang et al., 2018) and breast cancer (Kidokoro et al., 2008; Kim et al., 2012; Parmar et al., 2018).

RRM2 is a reductase that catalyzes the formation of deoxyribonucleotides from ribonucleotides. Synthesis of RRM2 is regulated in a cell cycle-dependent manner. RRM2 was reported to play an oncogenic role by protecting tumor cells from endogenous replication stress (Zhang et al., 2019), defects in genes in the DNA repair pathways (Mazzu et al., 2019), angiogenesis (Zhang et al., 2009), etc. Some recent studies also revealed that RRM2 participated in hepatocellular carcinogenesis (Wang et al., 2016; Wu et al., 2018b; Yang, Lin & Liu, 2020), consistent with our results.

MAD2L1 and BUB1B are proteins involved in cell mitosis. MAD2L1 was reported to exert oncogenic effects in gastric cancer (Wang et al., 2019b) and lung cancer (Guo et al., 2010), as well as HCC by regulating the malignant behaviors of tumor cells (Foijer et al., 2017; Li, Bai & Zhang, 2017). BUB1B was also reported to play a role in a series of cancers, such as colon cancer (Abal et al., 2007), brain tumor (Ding et al., 2013), glioblastoma (Lee et al., 2017) and breast cancer (Scintu et al., 2007). However, its mechanistic role in hepatocellular carcinogenesis remains unclear.

In this study, we further explored the role of BUB1B in HCC and its relationship with the other identified hub genes. We found that BUB1B was closely related to the other hub genes, and its expression was significantly higher in HCC cell lines than in normal hepatic cells. Our in vitro experiments indicate that BUB1B plays roles in HCC cell proliferation, migration, invasion, apoptosis and cell cycle. Further mechanistic studies showed that BUB1B exerts its oncogenic effect partially by affecting mitochondrial function. However, further studies based on larger sample sizes and a series of mechanistic experiments should be carried out to validate the present findings.

## Conclusions

We identified seven hub genes (BUB1B, CCNB1, CCNB2, CDC20, CDK1, MAD2L1 and RRM2) that were associated with the expression and prognosis of HCC based on different databases and a large number of clinical samples. And the oncogenic role of BUB1B in HCC was first explained by integrated bioinformatics analysis and in vitro experiments. BUB1B exerts its oncogenic effect partially by affecting mitochondrial function. In summary, our results provided a fundamental contribution for further researches aimed to find novel diagnostic or prognostic biomarkers as well as therapeutic targets for HCC.

##  Supplemental Information

10.7717/peerj.10943/supp-1 Supplemental Information 1 Fig. 9 raw data

10.7717/peerj.10943/supp-2 Supplemental Information 2 Fig. 10 raw data

10.7717/peerj.10943/supp-3 Supplemental Information 3 BUB1B overexpression promoted the proliferation, migration, invasion, cell cycle and inhibited the apoptosis of HCC cells (A) qRT-PCR was used to confirm the overexpression efficiency of the plasmid of BUB1B. (B, C) CCK-8 assays and colony formation assays were performed to evaluate the proliferation of the HCC cell lines. (D, E) The effects of BUB1B overexpression on cell migration and invasion were determined. (F, G) The cell apoptosis rate and cell cycle were analyzed by flow cytometry in HCC cells. HCC, hepatocellular carcinoma; CCK-8, Cell Counting Cit-8. ^∗^*P* < 0.05, ^∗∗^*P* < 0.01, ^∗∗∗^*P* < 0.001.

10.7717/peerj.10943/supp-4 Supplemental Information 4 BUB1B overexpression increased ATP content, OCR and mitochondrial membrane potential in HCC cells Total ATP production was detected in different treatment groups (A). Mitochondrial membrane potential was analyzed by JC-1 staining (B). The basal OCR of HCC cells were measured using an XF-24 analyzer (C). OCR, oxygen consumption rate; HCC, hepatocellular carcinoma. ^∗^*P* < 0.05, ^∗∗^*P* < 0.01, ^∗∗∗^*P* < 0.001.

10.7717/peerj.10943/supp-5 Supplemental Information 5 Fig. S1 raw data

10.7717/peerj.10943/supp-6 Supplemental Information 6 Fig. S1 raw data

## Additional Information and Declarations

### Data Availability

The following information was supplied regarding data availability: Sequences are available at NCBI GEO: GSE45267, GSE98383, GSE101685 and GSE112790. Raw data are available in the Supplemental Files.
