# Supplementary figures and images for: Identification of the hub gene BUB1B in hepatocellular carcinoma via bioinformatic analysis and in vitro experiments
Source: PeerJ. 2021 Feb 23;9:e10943. doi: 10.7717/peerj.10943 (PMC7908873; doi:10.7717/peerj.10943)

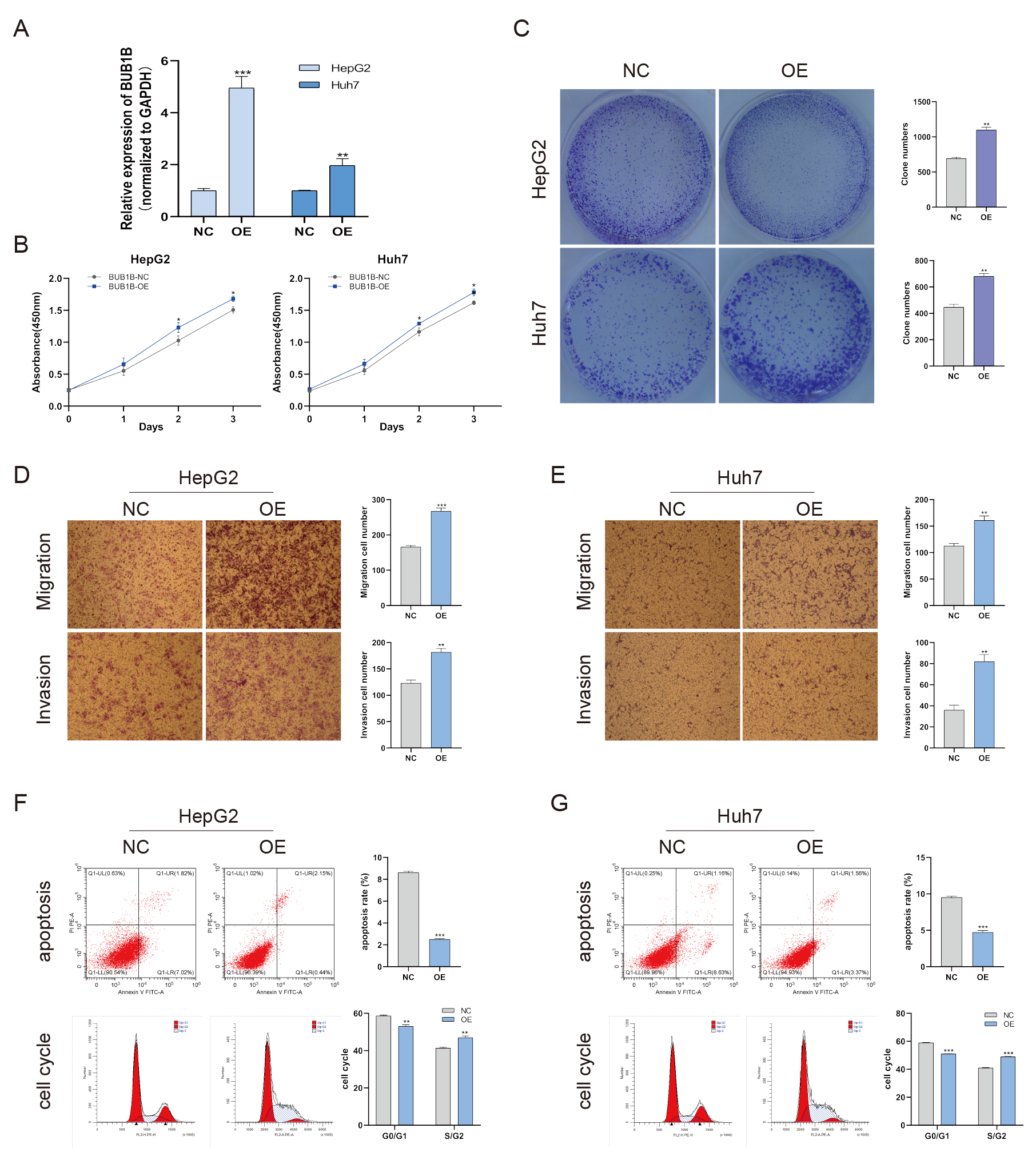

Supplement: Supplemental Information 3 — (A) qRT-PCR was used to confirm the overexpression efficiency of the plasmid of BUB1B. (B, C) CCK-8 assays and colony formation assays were performed to evaluate the proliferation of the HCC cell lines. (D, E) The effects of BUB1B overexpression on cell migration and invasion were determined. (F, G) The cell apoptosis rate and cell cycle were analyzed by flow cytometry in HCC cells. HCC, hepatocellular carcinoma; CCK-8, Cell Counting Cit-8. ∗P < 0.05, ∗∗P < 0.01, ∗∗∗P < 0.001. [file peerj-09-10943-s003.png]

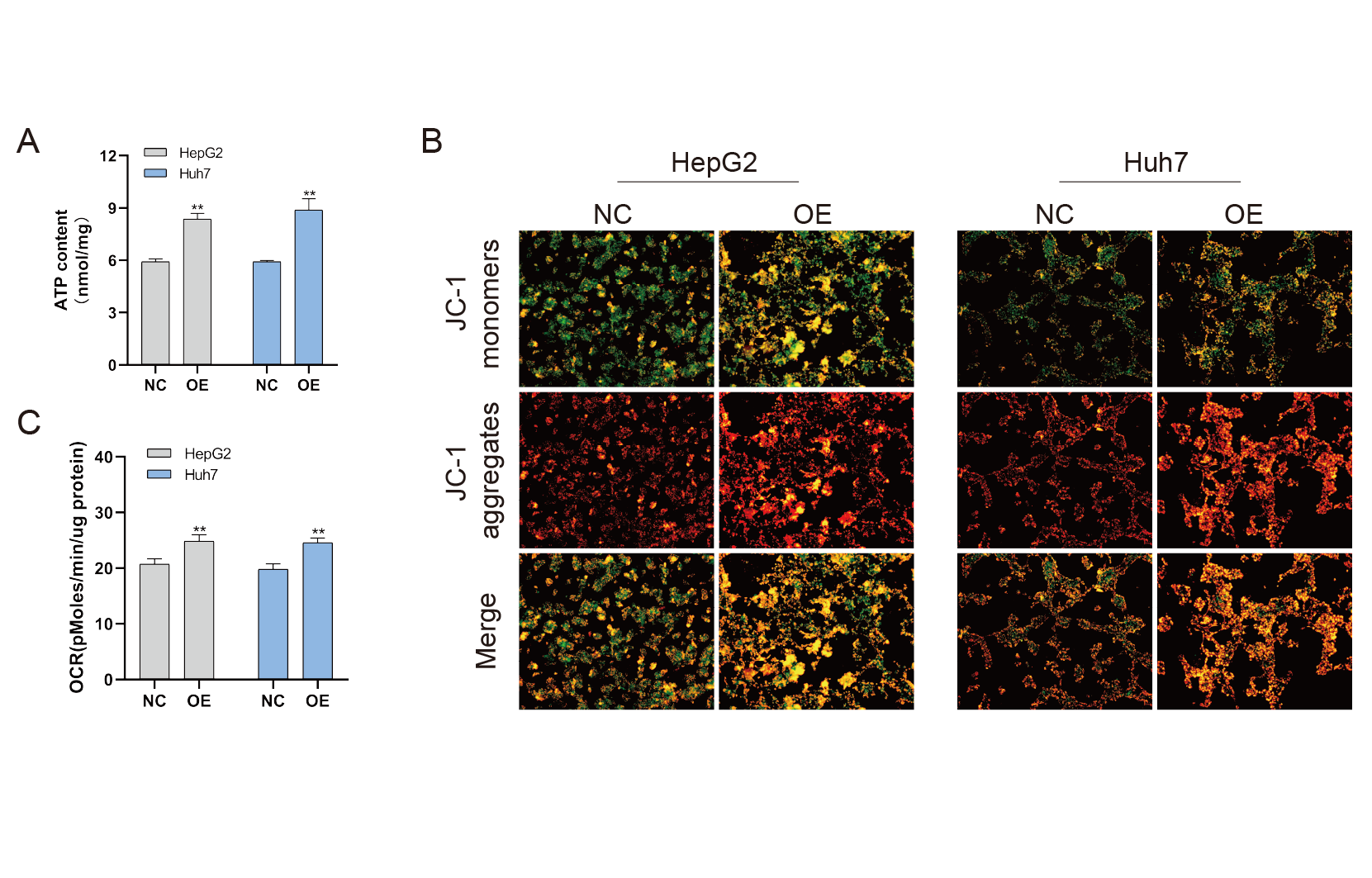

Supplement: Supplemental Information 4 — Total ATP production was detected in different treatment groups (A). Mitochondrial membrane potential was analyzed by JC-1 staining (B). The basal OCR of HCC cells were measured using an XF-24 analyzer (C). OCR, oxygen consumption rate; HCC, hepatocellular carcinoma. ∗P < 0.05, ∗∗P < 0.01, ∗∗∗P < 0.001. [file peerj-09-10943-s004.png]
